# Evaluation of the usefulness of insertion-null markers in critical skeletal remains

**DOI:** 10.1007/s00414-024-03205-3

**Published:** 2024-03-20

**Authors:** Christian Haarkötter, María Saiz, Xiomara Gálvez, Diana C. Vinueza-Espinosa, María Isabel Medina-Lozano, Juan Carlos Álvarez, Jose Antonio Lorente

**Affiliations:** Laboratory of Genetic Identification & Human Rights (LABIGEN-UGR), Department of Legal Medicine, Faculty of Medicine, University of Granada, Av. Investigación 11 – PTS – 18016, Granada, Spain

**Keywords:** Forensic Genetics, Human remains, INNULs, Retrotransposable elements, STRs

## Abstract

Forensic DNA analysis in compromised skeletal remains may pose challenges due to DNA degradation, often resulting in partial or negative autosomal STRs profiles. To address this issue, alternative approaches such as mitochondrial DNA or SNPs typing may be employed; however, they are labour-intensive and costly. Insertion-null alleles (INNULs), short interspersed nuclear elements, have been suggested as a valuable tool for human identification in challenging samples due to their small amplicon size. A commercial kit including 20 INNULs markers along with amelogenin (InnoTyper® 21) has been developed. This study assesses its utility using degraded skeletal remains, comparing the results obtained (the number of detected alleles, RFU values, PHR, and the number of reportable markers) to those obtained using GlobalFiler™. Subsequently, the random match probability of the two profiles for each sample was determined using Familias version 3 to evaluate the power of discrimination of the results obtained from each kit. In every sample, InnoTyper® 21 yielded more alleles, higher RFU values, and a greater number of reportable loci. However, in most cases, both profiles were similarly informative. In conclusion, InnoTyper® 21 serves as a valuable complement to the analysis of challenging samples in cases where a poor or negative profile was obtained.

**Supplementary Information:**

The online version contains supplementary material available at 10.1007/s00414-024-03205-3.

## Introduction

Short Tandem Repeats (STRs) represent a well-established method in forensic science for human identity testing applications [1]. However, forensic DNA analysis of degraded skeletal remains can be challenging due to DNA fragmentation and molecular damage resulting from decomposition and environmental exposure [2]. This phenomenon is linked to environmental factors such as temperature [3], humidity [4], salinity, and low pH values [5].

Commercial STR kits typically produce amplicons ranging from 100 to 500 base pairs (bp) [6], potentially resulting in partial or even negative profile due to DNA degradation. To address this issue, smaller STRs, known as miniSTRs, were developed by redesigning primer binding sites to reduce amplicon size [7]. Another approach for dealing with highly degraded sample is mitochondrial DNA (mtDNA) analysis, which involves the use of overlapping small-sized amplicons. However, this method is both labour-intensive and costly [8]. Similar challenges arise with single nucleotide polymorphisms (SNPs) [9].

Insertion and deletion polymorphisms fall somewhere between STRs and SNPs, and they offer the advantage of being compatible with routine capillary electrophoresis based workflows [10]. Retrotransposable elements (Res) include long and short interspersed nuclear elements (LINEs and SINEs respectively). Among SINEs, *Alu* sequences contain insertion and null alleles (INNULs) that differ in length [11]. A commercial kit comprising 20 INNULs markers, designed with a three-primers strategy (including a common forward primer for both alleles and a specific one for insertion and null alleles) was developed and marketed as InnoTyper® 21 [12].

INNULs typing offers several advantages, including: small amplicon size [13], absence of stutter artifacts [14], and a low mutation rate. However, a significant disadvantage is the substantial difference in length between insertion and null alleles, leading to a preferential amplification of the smaller one [15]. These characteristics make INNULs applicable in human identification [16], the analysis of degraded samples [17], the interpretation of mixtures [18], and population studies and biogeographical ancestry [19].

Our laboratory focuses on identifying the victims of the Spanish Civil War (1936–1939) and the postwar period in Andalusia -southern Spain- under an agreement between the Andalusia local government and our university. The remains we analyse here are highly degraded and often result in partial profiles. As a result, we continually explore alternative approaches to enhance the available information.

The aim of this research is to assess the performance of the InnoTyper® 21 commercial kit for INNULs in a large set of highly degraded skeletal remains samples and compare its efficiency to the autosomal STR kit Globalfiler™ while considering the discriminatory power of both kits.

## Material and methods

The following procedures were conducted in a low copy number DNA facility adhering to international standards for ancient DNA work [20–22]. This facility is equipped with measures to prevent contamination, including High Efficiency Particle Arresting (HEPA) filtered air positive pressure, a C type ultraviolet room for decontamination, DNAZap™ surfaces decontamination and the use sterile material. In addition to preventive measures, contamination detection protocols were implemented. These protocols involved identifying degraded DNA characteristics such as low quantification results, a high degradation index, and ‘ski-slope’ profiles. Furthermore, profiles of laboratory staff were regularly compared to those obtained from the samples.

### Samples

A total of 70 skeletal remains (see Table 1) retrieved from mass graves in Andalusia were analysed in this study. These samples were selected for comparison when a partial or negative Globalfiler™ profile was obtained. The remains had been buried at a depth of 3–4 m for 70–80 years in a region characterized by high temperatures in summer (an average 28ºC with maximums of 45ºC), over 2800 h of solar radiation, low precipitation (400–600 mm rain gauge on rare rainy days) [23], and slightly acidic soil [24].

**Table 1:** Samples by type of skeletal remain

	Femur	Tooth	Humerus	Tibia	Ulna
N	43	9	9	7	2

The surface of the samples was sanded with a Dremel® rotatory tool [25]. Subsequently, the bones were cut into fragments and exposed to UV light for 10 min each side in a 6 W cabin [26]. Tooth and bone fragments were pulverized using a TissueLyser II (QIAGEN, Hilden, Germany). The resulting tooth or bone powder was transferred to a 15-ml Falcon tube.

### DNA extraction

DNA from the samples was extracted using an in-house procedure based on the phenol/chloroform/isoamyl alcohol protocol, chosen for its ability to yield higher DNA amounts [27]. One gram of bone or tooth powder was mixed with 5 ml of lysis buffer containing EDTA, proteinase K, SDS (sodium dodecyl sulphate) and DTT (dithiothreitol), and then incubated at 56 ºC overnight. The lysate was mixed with phenol/chloroform/isoamyl alcohol (25:24:1) and the supernatant was concentrated using Amicon® Ultra-4 centrifugal filter unit (Merck, KGaA, Darmstadt, Germany). The extracts were purified using the MinElute® PCR Purification Kit (QIAGEN, Hilden, Germany).

### DNA quantification

The purified extracts were quantified by the Quantifiler™ Trio quantitative PCR commercial kit following the manufacturer’s instructions [28]. The qPCR reaction was carried out in a QuantStudio® 5 (ThermoFisher).

### DNA amplification

The DNA extracts were amplified using the Globalfiler™ autosomal STR commercial kit (ThermoFisher, Waltham, MA, USA), and the Innotyper® 21 INNULs commercial kit (InnoGenomics, New Orleans, LA) following the manufacturer’s recommendations in both cases [29, 30]. The same amount of DNA extract (15 µl) was added to each reaction.

### Data analysis

The amplified samples were subsequently analysed using a 3500 Genetic Analyzer, following the injection parameters provided by each manufacturer. The raw data was analysed using GeneMapper™ IDX v1.6. Four parameters were analysed: 1) the number of detected alleles (alleles above the analytical threshold of 50 RFU for Globalfiler™, and 85 RFU for InnoTyper® 21), 2) average RFU (relative fluorescence units), 3) average PHR (peak height ratio, calculated as the ratio of the smaller allele to the larger allele in heterozygous loci), and 4) the number of reportable loci (markers with homozygous alleles above stochastic threshold, 360 RFU for Globalfiler™ and 180 RFU for InnoTyper® 21, as well as markers with heterozygous alleles above analytical threshold and PHR above 0.60 for Globalfiler™ and 0.76 for InnoTyper® 21). All thresholds were established following internal validation in accordance with SWGDAM’s guidelines [31].

Statistical parameters (mean, coefficient of quartile variation (CQV), Shapiro–Wilk test for normality, Levene’s test for homogeneity of variances, and One-Way ANOVA, including both Welch’s and Fisher’s, were calculated using jamovi 2.2.5 [32].

To evaluate the power of discrimination of each obtained profile, random match probability (RMP) was calculated using Familias version 3 [33]. The calculations utilized 2023 GHEP-ISFG STRs allele frequencies [34] and an earlier publication regarding InnoTyper® 21 allele frequencies in the Andalusian population [19].

## Results and discussion

The results of the genetic profiles are summarised in Table 2, presenting both average values and data dispersion as the coefficient of quartile variation. More than 90% of the samples yielded low quantification results for the small DNA target (refer to Supplementary Data), and approximately 85% of the samples yielded five or fewer reportable autosomal STR markers. Within this subset (5 informative markers or less), 32% of them resulted in a negative profile (with no reportable markers). The best Globalfiler™ profile (14 markers) was obtained with a total of 0.738 ng of DNA input, while the best InnoTyper™ result (19 markers) was obtained with a total of 0.091 ng of DNA input, highlighting the higher sensibility of the latter.

**Table 2:** Average and coefficient of quartile variation (CQV) of the detected alleles (above the analytical threshold), relative fluorescence units (RFU), peak height ratio (PHR), and reportable loci by kit

Kit		Detected alleles	$\overline{{\text{RFU}} }$	PHR	Reportable loci
Globalfiler™	x̄	12	207	0.47	3
	CQV	0.648	0,429	1.000	1.000
InnoTyper® 21	x̄	20	567	0.49	8
	CQV	0.415	0.511	0.277	0.625

**Fig. 1:**
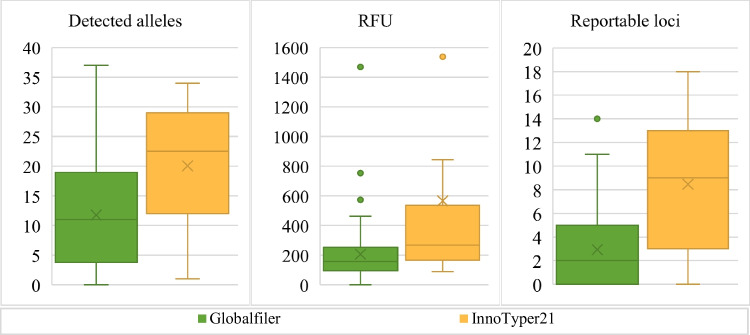
Boxplots of detected alleles, average relative fluorescence units, and reportable loci by kit

In Fig. 1, boxplots depict the analysed variables of genetic profiles. In general terms, InnoTyper® 21 outperforms Globalfiler™ in the number of detected alleles and RFU, reaching approximately twice as many in both cases. Peak height ratios were nearly identical in both kits. Concerning the number of reportable loci, InnoTyper® 21 tripled the count.

**Fig. 2:**
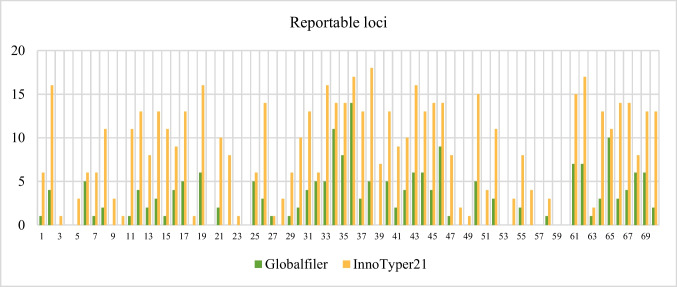
Bar chart showing the number of reportable markers obtained by each sample using each commercial kit

Figure 2 illustrates the number of reportable markers obtained in each sample by kit. Out of the 22 samples that failed to produce reportable markers, 15 of them yielded a positive result with InnoTyper® 21, although they were mostly partial profiles. In many cases, when 5 markers or fewer were obtained by Globalfiler™, InnoTyper® 21 managed to achieve at least half of the profile.

After conducting Shapiro–Wilk and Levene tests, One-Way ANOVA revealed statistically significant differences between the two kits concerning the number of detected alleles (p-value < 0.001), average RFU (p-value = 0.003), and the number of reportable loci (p-value < 0.001). No statistically significant differences were found in terms of peak height ratio (p-value = 0.808).

Previous studies have indicated that InnoTyper® 21 detected more alleles than autosomal STR kits like NGM™ [35] and Globalfiler™ [12, 17, 36], resulting in superior profiles [17, 36], and informative profiles even when Globalfiler™ yielded zero markers [12, 17]. Our findings align with these results, as InnoTyper® 21 obtained more detected alleles and a higher number of reportable markers. However, the results from skeletal remains with prior Gobalfiler™ negative profiles are not as informative as the InnoTyper™ 21 profiles in the study conducted by Martins et al. [17]. These differences may be attributed to their samples being casework rootless hairs.

Nevertheless, there are limited examples in the published literature where not only the number of markers reached by each autosomal STRs/INNULs kit is discussed, but also the statistical significance of both profiles. This evaluation is crucial to determine if InnoTyper® 21 results hold enough power of discrimination for potential reporting.

**Fig. 3:**
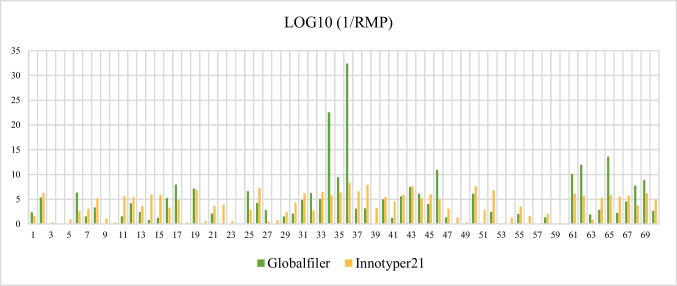
Bar chart illustrating the common logarithm of the likelihood ratio values (1/RMP) obtained by each sample using each commercial kit

To assess this, the random match probability (RMP) was calculated using Familias version 3 software for each Globalfiler™^/^InnoTyper® 21 kit. Figure 3 displays the common logarithm (base 10) of each profile by sample and by commercial kit.

When comparing both Figs. 2 and 3, it is evident that InnoTyper® 21 yields more markers. Nevertheless, its power of discrimination aligns with that already achieved by GlobalFiler™. In fact, no statistically significant differences were observed between the two groups of likelihood ratios (p-value = 0.321) in One-Way ANOVA.

InnoGenomics tested InnoTyper® 21 with sonicated samples and concluded that higher random match probability values were achieved when DNA fragments were < 150 bp compared to the values obtained by Globalfiler™ [37]. Our research supports this idea, as InnoTyper® 21 outperforms Globalfiler™ in terms of random match probability when fewer than 5 markers were achieved with the autosomal STRs commercial kit. However, it is true that the power of discrimination of GlobalFiler™ is astronomical compared to InnoTyper® 21 when 10 or more markers are achieved by the former.

This is why many authors suggests InnoTyper® 21 as a valuable complement to autosomal STRs [12, 35, 38]. Some even propose that the power of discrimination offered by InnoTyper® 21 is higher than the provided by mitochondrial DNA [17]. This is especially true when little markers or a negative profile have been achieved with autosomal STRs approaches.

One last point to consider is whether both likelihood ratios obtained from Globalfiler™ and InnoTyper® 21 can be combined. This topic has been widely discussed in literature, with two main positions: one advocating for the avoidance of combining different DNA evidence [39], and the other one supporting the combination using the product rule [40], with the associated mathematical refinement over time [41]. The key factor is demonstrating the independence between autosomal STRs and INNULs, a question that remains unanswered in the literature. However, a roughly calculated Kosambi recombination fraction [42] gives extremely low values to the markers located in the same loci: AC4027 and D7S820, TARBP and D1S1656, and NBC106 and FGA.

## Conclusion

The aim of this research was to evaluate the applicability of InnoTyper® 21 for the analysis of degraded skeletal remains’ DNA and to estimate the power of discrimination obtained from partial INNULs profiles compared to an autosomal STRs approach. A total of 70 degraded skeletal remains samples were typed with Innotyper® 21, specifically selected when a negative or partial profile was obtained by Globalfiler™^.^ InnoTyper® 21 consistently yielded more alleles, higher RFU values, and more reportable loci than Globalfiler™ in every sample. However, despite these differences in profiling, the random match probability values from both profiles were similar. In conclusion, InnoTyper® 21 emerges as a robust complementary tool for addressing partial or negative results in challenging samples.

### Supplementary Information

Below is the link to the electronic supplementary material.

Supplementary file1 (XLSX 17 KB)

## Data Availability

The data supporting the findings of this study are available within the paper and its Supplementary Information.
